# Enzymatic synthesis of bioactive compounds with high potential for cosmeceutical application

**DOI:** 10.1007/s00253-016-7647-9

**Published:** 2016-06-08

**Authors:** Io Antonopoulou, Simona Varriale, Evangelos Topakas, Ulrika Rova, Paul Christakopoulos, Vincenza Faraco

**Affiliations:** Division of Chemical Engineering, Department of Civil, Environmental and Natural Resources Engineering, Luleå University of Technology, 97187 Luleå, Sweden; Department of Chemical Sciences, University of Naples “Federico II”, Naples, Italy; Biotechnology Laboratory, School of Chemical Engineering, National Technical University of Athens, 15700 Athens, Greece

**Keywords:** Lipases, Feruloyl esterases, Tannases, Transferases, Glycosidases, Proteases, Laccases, Anti-oxidant, Anti-microbial, Anti-inflammatory, Skin whitening, Anti-wrinkling, Anti-aging, Photoprotective, Fungal, Bacterial

## Abstract

Cosmeceuticals are cosmetic products containing biologically active ingredients purporting to offer a pharmaceutical therapeutic benefit. The active ingredients can be extracted and purified from natural sources (botanicals, herbal extracts, or animals) but can also be obtained biotechnologically by fermentation and cell cultures or by enzymatic synthesis and modification of natural compounds. A cosmeceutical ingredient should possess an attractive property such as anti-oxidant, anti-inflammatory, skin whitening, anti-aging, anti-wrinkling, or photoprotective activity, among others. During the past years, there has been an increased interest on the enzymatic synthesis of bioactive esters and glycosides based on (trans)esterification, (trans)glycosylation, or oxidation reactions. Natural bioactive compounds with exceptional theurapeutic properties and low toxicity may offer a new insight into the design and development of potent and beneficial cosmetics. This review gives an overview of the enzymatic modifications which are performed currently for the synthesis of products with attractive properties for the cosmeceutical industry.

## Introduction

Articles defined as cosmetics are intended for human body application aiming at increased beauty and attraction or cleaning use, without affecting the body structure or function (Nelson and Rumsfield 1988). During the last few years, the cosmetic industry is searching for bioactive compounds that also promote health benefits. This combination resulted in a new term called “cosmeceutical” where cosmetic products assert medical benefits (Choi and Berson 2006). Cosmeceuticals are different from cosmetics and drugs, as they affect the function and structure of skin, while having drug-like effects that are marketed using skin appearance-based claims. Cosmeceutical industry numbers over 400 manufacturers worldwide including Estée Lauder, L’Oréal, Procter & Gamble, and Avon, with 80 % of the US and European market dedicated to skin care (Brandt et al. 2011). In 2008, Japan was by far the biggest market in cosmeceuticals valued at $6–8 billion, followed by the USA ($5–6 billion) and EU ($3–5 billion) (Kim and Wijesekara 2012). Market growth is expected to rise in economies like China, Brazil, the Russian Federation, and India (Brandt et al. 2011). Nevertheless, the Food and Drug Administration (FDA) does not recognize cosmeceutical as a term even if it is widely used in industry, while in the EU, most are considered as cosmetics (Sharma 2011). There is no regulation of cosmeceuticals in EU, the USA, and Japan; however, as the interaction between cosmetic and skin is complex, there is an increased attention towards the need of toxicological tests of the final product and its bioactive ingredients (Nohynek et al. 2010). Target ingredients of cosmeceuticals may include phytochemicals, vitamins, peptides, enzymes, essential oils among others, which are incorporated into lotions, creams, and ointments dedicated to skin treatment. Desired properties, such as anti-oxidant, anti-aging, anti-microbial, anti-wrinkling, photoprotective, or skin whitening, are preferentially offered by natural compounds derived from plant or sea organisms, instead of chemically synthetic compounds. The guidelines of the Council of Europe define a natural cosmetic as a product that consists of natural substances of botanical, mineral, or animal origin, exclusively obtained through physical, microbiological, or enzymatic methods, with certain exceptions for fragrances and preservatives. This demand has increased the sales of personal care products based on natural ingredients; however, often a modification of the bioactive compounds is required prior to their application in the final product, e.g., by increasing its lipophilicity or improving its biological properties. Modification with fatty compounds generally results in more lipophilic products, whereas modification with sugars results in more hydrophilic derivatives. Chemical approaches have numerous disadvantages such as the protection and de-protection of groups resulting in many reaction steps, use of strong acid as catalyst, high temperatures (150–200 °C), formation of unwanted products, dark color, burnt taste of product, and high energy consumption (Kiran and Divakar 2001). Enzymatic modification is employed under mild conditions, is highly selective, and includes one single step.

**Fig. 1:**
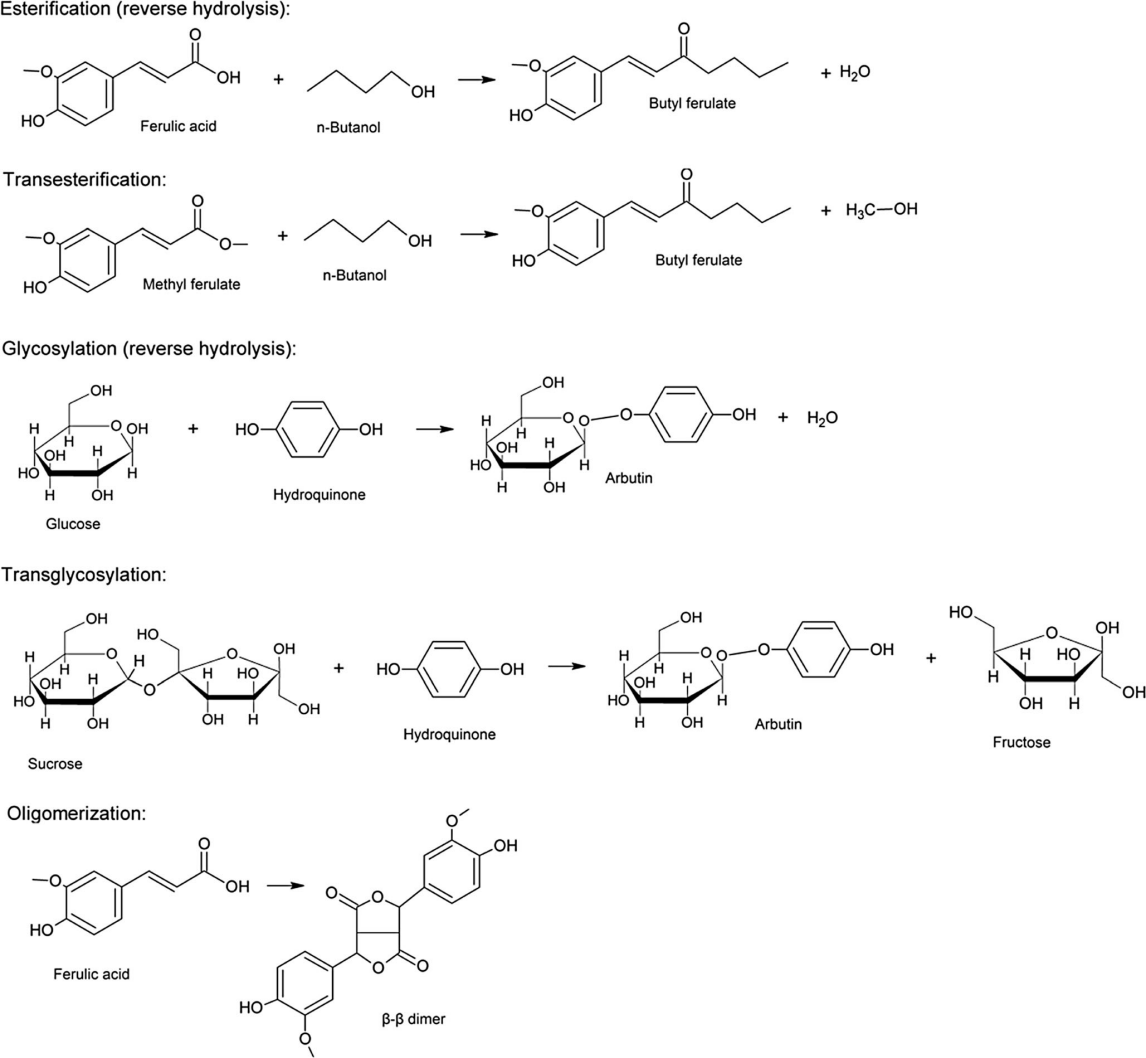
Reaction examples

In this review, the most important enzymatic modifications that result to the synthesis of ingredients with attractive properties for the cosmeceutical industry are documented. Properties such as anti-oxidant, anti-inflammatory, anti-microbial, skin-whitening, and photoprotective effects were criteria for the selection of the reported modification reactions. A modification may follow different mechanisms: direct esterification or transesterification performed by esterases (such as lipases, feruloyl esterases, or tannases) and proteases, glycosylation (reverse hydrolysis) or transglycosylation performed by transferases, and β-glucosidases and oligomerization performed by laccases. Examples of such modification reactions are presented in Fig. 1.

## Esterases

Except for their hydrolytic ability, esterases are able to perform (trans)esterification reactions. Triaglycerol lipases (EC 3.1.1.3) are most commonly used due to their broad specificity, as shown in Table 1. Less popular, ferulic acid esterases (FAEs; EC 3.1.1.73) generally catalyze the hydrolysis of the ester bond between the main chain polysaccharides of xylans or pectins and the monomeric or dimeric ferulic acid in plants; however, they are able to modify hydroxycinnamic acids and their esters. Tannases (tannin acyl hydrolases, EC 3.1.1.20) are known to be active on complex polyphenolics, catalyzing the hydrolysis or synthesis of the “ester bond” (galloyl ester of an alcohol moiety) or the “depside” bond (galloyl ester of gallic acid) (Battestin et al. 2008). Low water content is essential for the thermodynamic shift of equilibrium towards synthesis. Different systems have been employed including organic co-solvents, ionic liquids, solvent-free systems, supercritical fluids, and molecular sieves as water removal agents. The ideal solvent should aid solubilization of substrates, not affect enzyme activity, have low toxicity, and enable easy product recovery (Wei et al. 2002). Ionic liquids are a good alternative since they generally do not deactivate esterases and have exceptional tailorability and low volatility (Zeuner et al. 2011). However, a number of issues including the cost involved in large-scale usage are to be addressed. Aids as microwave irradiation and ultrasound treatment have been employed in lipase-catalyzed reactions (Costa et al. 2014; Cui et al. 2013). Detergentless microemulsions, so far employed in FAE-catalyzed reactions, consist of a hydrocarbon, a short-chained alcohol, and water representing thermodynamically stable dispersions of aqueous microdroplets in the hydrocarbon solvent (Khmelnitsky et al. 1988). An important advantage of these mixtures is the separation of reaction products and enzyme reuse, while the solubility of relatively polar phenolic acids is high owing to the presence of large amount of polar alcohol.

**Table 1:** Lipase-catalyzed reactions

Product	Donor	Acceptor	Enzyme	Solvent system	Yield (time)	T (°C)	Reference
Examples of α-hydroxy acid derivatives							
C6–C18 lactates	C6–C18 fatty alcohols	Lactic acid	Novozym 435	Acetonitrile	94–96 % (48 h)	30	Torres and Otero 1999
C6–C18 glycolates	C6–C18 fatty alcohols	Glycolic acid	Novozym 435	Hexane	91 % (48 h)		
Ethyl glycoside lactate	Ethyl glycoside	Butyl lactate	Novozym 435	Solvent-free	95 % (36 h)	60	Wei et al. 2002
β-Methyl glycoside malate/glycolate/lactate	β-Methyl glycoside	Malic/glycolic/lactic acid	Novozym 435	*t*-Butanol	48–75 % (120 h)	60	Park et al. 2001
Palmitoyl or stearoyl lactic acid	C16 or C18:0 fatty acid	Lactic acid	Lipozyme IM20	Ethyl methyl ketone	37.5–40 % (72 h)	37 or 60	Kiran and Divakar 2001
Examples of kojic acid derivatives							
Kojic acid monoricinoleate	Ricinoleic acid	Kojic acid	Lipozyme TL IM	Solvent-free	87.4 % (6 h)	80	El-Boulifi et al. 2014
Kojic acid monooleate	Oleic acid	Kojic acid	Amano G	Acetonitrile	36.7 % (48 h)	50	Liu and Shaw 1998
Kojic acid monopalmitate	Palmitic acid	Kojic acid	RM IM	Acetonitrile	29.30 % (12 h)	50	Lajis et al. 2013
Examples of lipoic acid derivatives							
Pyridoxine-O-lipoate (5′ and 4′)/tyrosol-8-O-lipoate/tyramin-8-N-lipoate	Pyridoxine (vitamin B6)	Lipoic acid	CNTs-C6-NH2-CaLB or CNTs-C11-CH3-CaLB	(mtoa)NTf2	91.1–99.5 % (72 h)	60	Papadopoulou et al. 2013
	Tyrosol/tyramine			(bmim)PF6			
Phenolic lipoates	4-Hydroxybenzyl alcohol/vanillyl alcohol/4-hydroxyphenyl ethanol/coniferyl alcohol/dihydroxybenzyl alcohol/dihydroxyphenyl ethanol	Lipoic acid	Novozym 435	2-Butanone: hexane	64–80 % (15 h)	25	Kaki et al. 2012
Octanyl lipoate	n-Octanol	α-Lipoic acid	Whole-cell lipase from *Aspergillus oryzae* WZ007	Heptane	75.2 % (48 h)	50	Yang et al. 2009
Examples of arbutin derivatives							
Arbutin lipoate	α-Lipoic acid	β-Arbutin	Type B lipase from *C. antarctica*	*t*-Butanol	– (7 days)	55	Ishihara et al. 2010
C2–C18 alkyl arbutin esters	Vinyl esters of C2–C18 aliphatic alcohols	β-Arbutin	Immobilized lipase from *Penicillium expansum*	Anhydrous THF	82–99 % (0.5–72 h)	35	Yang et al. 2010a
Arbutin phenolic acid esters	Vinyl esters of aromatic acids				30–99 % (4–96 h)	50	Yang et al. 2010b
Arbutin fatty acid esters	Saturated fatty acids (C6–C18)	β-Arbutin	Chirazyme L-2 C2	Acetonitrile	Up to 45 % (2 days)	60	Nagai et al. 2009
Arbutin ferulate	Ferulic acid	β-Arbutin	Type B lipase from *C. antarctica*	*t*-Butanol	57 % (7 days)	55	Ishihara et al. 2010
	Vinyl ferulate	p-Arbutin	Novozym 435	Acetonitrile	50 % (−)	45	Chigorimbo-Murefu et al. 2009
Examples of vitamin derivatives							
L-Ascorbyl palmitate	Palmitic acid	L-Ascorbic acid	Lipase from *Bacillus stearothermophilus* SB1	Hexane	97 % (6 h)	50	Bradoo et al. 1999
	Methyl palmitate		Lipase from *Burkholderia multivoras*	Solvent-free (under microwave irradiation)	83 % (40 min)	80	Kidwai et al. 2009
	Ethyl palmitate		Lipozyme TL IM	*t*-Butanol	20 % (120 h)	40	Reyes-Duarte et al. 2011
	Vinyl palmitate				100 % (120 h)		
	Tripalmitin				50 % (140 h)		
L-Ascorbyl oleate	Oleic acid	L-Ascorbic acid	Novozym 435	*t*-Amyl alcohol	82 % (52 h)	65	Viklund et al. 2003
	Methyl oleate		Lipozyme TL IM	*t-*Butanol	50 % (−)	60	Reyes-Duarte et al. 2011
	Triolein		Novozym 435	*t*-Amyl alcohol	84 % (140 h)	40	Moreno-Perez et al. 2013
	Olive oil		Novozyme 435-PEI		85 % (48 h)	45	
Conjugated linoleoyl ascorbates	C9t11CLA	L-Ascorbic acid	Chirazyme L-2 C3	Acetone	~80 % (~48 h)	50	Watanabe et al. 2008
L-Ascorbyl benzoate	Benzoic acid	L-Ascorbic acid	Novozym 435	Cyclohexanone	47.7 % (48)	66.6	Lv et al. 2008
L-Ascorbyl acetate	Vinyl acetate	L-Ascorbic acid	Lipozyme TLIM	Acetone	99 % (4)	40	Zhang et al. 2012
Vitamin E succinate	Succinic anydride	Rac-all-α-tocopherol	Succinyl-Novozym 435	DMSO:*t*-butanol	94.4 % (48 h)	40	Yin et al. 2011
Vitamin E acetate	Vinyl acetate	δ-Tocopherol	Novozym 435	*t*-Amyl alcohol	65 % (16 days)	60	Torres et al. 2008b
		α-Tocopherol			>45 % (16 days)		
Vitamin E ferulate	Ethyl ferulate	Vitamin E	Novozym 435	Solvent-free	25.2 % (72 h)	60	Xin et al. 2011
Sugar or ascorbyl retinyl adipates	Sorbitol/fructose/glucose/saccharose/maltose/ascorbic acid	Retinyl adipate	Novozym 435	*t*-Amyl alcohol	22–80 % (45 h)	40	Rejasse et al. 2003
Vitamin A lactate	Lactic acid	Vitamin A acetate	Immobilized lipase from *C. antarctica*	Hexane	32 % (7 h)	30	Liu et al. 2012
	Methyl lactate	Retinol	Lipozyme		86 % (50 h)	55	Maugard and Legoy 2000
Vitamin A oleate	Methyl oleate			Hexane	90 % (50 h)		
	Oleic acid	Retinyl acetate	Immobilized lipase from *C. antarctica*		79 % (7 h)	30	Liu et al. 2012
Vitamin A saturated fatty acid esters (C6–C18)	C6–C18 saturated fatty acids			Hexane	51–82 % (7 h)		
Vitamin A methyl succinate	Dimethyl succinate	Retinol	Lipozyme	Hexane	77 % (50 h)	55	Maugard and Legoy 2000
Examples of flavonoid derivatives							
Quercetin derivatives	C18–C12 fatty acids	Isoquercetin	Novozym 435	Acetone or acetonitrile	81–98 % (18–24 h)	45–60	Ziaullah 2013
	Ethyl esters of C4–C18 fatty acids		Novozym 435	*t*-Amyl alcohol	38–66 % (72 h)	65	Salem et al. 2010
	Cinnamic acids		Novozym 435	*t*-Butanol	17–89 % (7 days)	60	Stevenson et al. 2006
	Dibenzyl malonate		Lipase from *C. antarctica*	Me_2_CO: pyridine	74 % (12 days)	45	Riva 1996
	Vinyl acetate		PSL-C II	Acetone	84 % (96 h)	50	Chebil et al. 2007
		Quercetin			100 % (24 h)		
Silybin derivatives	Divinyl ester of decanoic acid	Silybin	Novozym 435	Acetonitrile	26–66 % (72 h)	45	Vavrikova et al. 2014
	Vinyl butanoate		Novozym 435	Acetone	100 % (24–96 h)	50	Theodosiou et al. 2009
	Vinyl acetate		Novozym 435	Acetone	92 % (48 h)	35	Gazak et al. 2010
Esculin derivatives	Fatty acids, dicarboxylic acids, other cyclic acids	Esculin	Novozym 435	*t*-Amyl alcohol	13–90 % (12 h)	60	Ardhaoui et al. 2004a
	Palmitic acid		Novozym 435	TOMA TF2N	>96 % (6 days)	60	Lue et al. 2010
	Vinyl butyrate		Novozym 435	[Bmim]BF6	90.6 % (72 h)	60	Katsoura et al. 2007
Phloridzin derivatives	C2–C18 fatty acids	Phloridzin	Novozym 435	Acetonitrile	70–90 % (7 days)	65	Milisavljecic et al. 2014
	Ethyl cinnamate		Novozym 435	Solvent-free	100 % (4 h)	80	Enaud et al. 2004
Hesperedin derivatives	Decanoic acid	Hesperidin	Novozym 435	[Bmim]BF4: acetone	53.6 % (96 h)	50	Branco de Araujo et al. 2011
	Palmitic acid		Novozym SP435	*t*-Amyl alcohol	Up to 40 % (12 h)	60	Ardhaoui et al. 2004b
	Vinyl acetate	Hesperetin	PSL-C II	Acetonitrile	30 % (96 h)	50	Chebil et al. 2007
Rutin derivatives	C4–C18 fatty acids	Rutin	CALB	*t*-Amyl alcohol	27–62 % (168 h)	60	Viskupicova et al. 2010
	Ethyl linoleate		Novozym 435	Acetone	50 % (96 h)	50	Mellou et al. 2006
	Methyl palmitate		Novozym 435	*t*-Amyl alcohol	30 % (48 h)	60	Passicos et al. 2004
	Vinyl esters of fatty acids		Novozym 435	[Bmim]BF4	15–65 % (96 h)	60	Katsoura et al. 2006
	Dicarboxylic acids, fatty acids, other cyclic acids		Novozym 435	*t-*Amyl alcohol	10–90 % (−)	60	Ardhaoui et al. 2004a
	Divinyl dicarboxylate		Novozym 435	*t*-Butanol	36 % (4 days)	50	Xiao et al. 2005
	Dibenzyl malonate		Lipase from *C. antarctica*	Me2CO: pyridine	79 % (12 h)	45	Riva 1996
	Vinyl cinnamate		Chirazyme L-2	Acetone	28 % (14 h)	37	Ishihara et al. 2002
Naringin derivatives	α-Linolenic acid, linoleic, oleic acid	Naringin	Novozym 435	*t*-Amyl alcohol	83.2–90.1 % (72 h) (assisted by ultrasound irradiation)	50	Zheng et al. 2013
	Stearic acid		Novozym 435	*t*-Amyl alcohol	46 % (24 h)	60	Duan et al. 2006
	Vinyl butyrate		Novozym 435	[Bmim]BF_4_	86.9 % (100 h)	60	Katsoura et al. 2007
	Methyl palmitate		Novozym 435	*t*-Amyl alcohol	92 % (48 h)	60	Passicos et al. 2004
	C10–C12 vinyl esters of saturated fatty acids		Novozym 435	Acetone	22–70 % (96 h)	50	Mellou et al. 2005
	PUFA from byfish products		Novozym 435	*t*-Amyl alcohol	30 % (96 h)	50	Mbatia et al. 2011
	Vinyl laurate		Lipozyme IM TL	*t*-Amyl alcohol	90 % (30 min)	52	Luo et al. 2013
	Lauric acid		Chirazyme L-2 C2	Acetonitrile	~45 % (~30 h)	60	Watanabe et al. 2009
	Ricinoleic acid		Immobilized lipase from *C. antarctica*	Acetone	24 % (120 h)	50	Almeida et al. 2012
	Castor oil				33 % (120 h)		
	Vinyl cinnamate		Chirazyme L-2	Acetone	64 % (14 days)	37	Ishihara et al. 2002
	Dibenzyl malonate		Lipase from *C. antarctica*	Acetone: pyridine	69 % (12 days)	45	Riva 1996
	Vinyl acetate	Naringenin	PSL-C II	Acetonitrile	100 % (96 h)	50	Chebil et al. 2007
Examples of hydroxycinnamic acid derivatives							
Feruloylated lipids	Ferulic acid	Glycerol	Chirazyme L2 C-2	Solvent-free	80 % (>3 h)	80	Matsuo et al. 2008
		Trilinolenin	Novozym 435	Hexane:2-butanone	14 % (5 days)	55	Sabally et al. 2006
		Flaxseed oil	Novozym 435	SCCO_2_ medium	57.6 % (27.5 h)	80	Ciftci and Saldana 2012
	Ethyl ferulate	Oleyl alcohol	Novozym 435	Hexane	99.17 % (4 days)	60	Chen et al. 2011b
		Triolein	Novozym 435	Toluene	77 % (144 h)	60	Compton et al. 2000
		Olive oil	Novozym 435	2M2B: toluene	59.6 % (2.34 h)	60	Radzi et al. 2014
		Tributyrin	Novozym 435	Solvent-free	94.2 % (120 h)	50	Zheng et al. 2008
		Monostearin	Novozym 435	Ethanol	97.8 % (23 h)	74	Sun et al. 2013a, b
		Soybean oil	Novozym 435	Glycerol	70 % (140 h)	60	Laszlo and Compton 2006
		Fish oil	Novozym 435	Toluene	80.4 % (5 days)	70	Yang et al. 2012
		Phosphatidylcholine	Novozym 435	Chloroform	40.51 % (4 days)	55	Yang et al. 2013
		Glycerol	Novozym 435	EMIMTF2N	100 % (12 h)	70	Sun et al. 2013a, b
		Oleic acid	Novozym 435	Solvent-free	96 % (1.33 h)	60	Sun et al. 2007
	Glyceryl ferulate	Oleic acid	Novozym 435	[Bmim]PF6	100 % (3 h)	80	Sun et al. 2009
	Vinyl ferulate	Triolein	Novozym 435	Solvent-free	91.9 % (62 h)	55	Yu et al. 2010
Methyl caffeate	Caffeic acid	Methanol	Novozym 435	[Bmim][Tf_2_N]	99.79 % (9 h)	75	Wang et al. 2015
Propyl caffeate	Methyl caffeate	1-Propanol	Novozym435	[Bmim][CF_3_SO_3_]	99.5 % (2.5 h)	60	Wang et al. 2013
Sitosteryl hydroxycinnamates	Vinyl ferulate/caffeate/sinapate	Sitosterol	Lipase type VII from *Candida rugosa*	Hexane:2-butanone	30–90 % (−)	45	Tan and Shahidi 2011; Tan and Shahidi 2012; Tan and Shahidi 2013
Examples of galloyl derivatives							
Propyl gallate	Gallic acid	1-Propanol	Immobilized lipase from *Staphylococcus xylosus*	Hexane	90 % (6 h)	52	Bouaziz et al. 2010
Mono-, di-, and tri- acetylated EGCG	Vinyl acetate	EGCG	Lipozyme RM IM	Acetonitrile	87.37 % (1.13 h)	40	Zhu et al. 2014
Catechin *5*-O and *7*-O acetate	Vinyl acetate	Catechin	PCL	Acetonitrile	70 % (48 h)	45	Lambusta et al. 1993

Novozym 435: lipase B from *Candida antarctica* immobilized on a macroporous acrylic resin (CALB); lipozyme IM20/lipozyme RM IM: lipase from *Rhizomucor miehei* immobilized on duolite anion exchange resion; lipozyme TL IM: lipase from *Thermomyces laniginosus* immobilized on silica granulation; amano G: lipase from *Penicillum camemberti*; CNTs-C_6_-NH_2_-CaLB, CNTs-C_11_-CH_3_-CAL-B: novozym 435 functionalized with various multi-walled carbon nanotube groups; chirazyme L-2: immobilized lipase B from *C. Antarctica*; succinyl-novozym 435: novozym 435 modified with succinic anhydride; PSL-C II, PCL: lipase from *Pseudomonas cepacia*

### α-Hydroxy acid derivatives

α-Hydroxy acids (AHAs) are composed of carbon backbones containing a carboxyl group and a hydroxyl group on the adjacent carbon. Among them, glycolic acid, lactic acid, and malic acid have been well known in cosmetics as beauty aids and peeling agents due to their hygroscopic, emulsifying, and exfoliating properties (Tung et al. 2000). Short-chain AHAs as lactic acid are more active in regulating the rate of skin regeneration and improving dryness (Wei et al. 2002). However, limiting factors for application are their acidicity and the rapid penetration into the deep epiderm, causing irritant effects at concentrations >10 %. To control their concentration and penetration to the skin’s intercellular spaces, AHAs have been grafted onto alkylglycosides, fatty acids, or fatty alcohols so they can be gradually released by the epidermis esterases. Short-chain alkylglycosides have been reported to relieve the irritant effects on skin after UV radiation (Wei et al. 2003). A major concern regarding enzymatic modification is that lactic acid can undergo self-polymerization at high temperatures and low water content forming linear polyesters or lactones because of the presence of groups that act as acyl donor and nucleophile at the same time (Roenne et al. 2005). A key factor is the choice of enzyme that favors the desired reaction. Lactic acid does not act as nucleophile when the lipase B from *Candida antarctica* (CALB) is used as biocatalyst due to steric hindrance at the enzyme’s active site (Form et al. 1997). Another obstacle is the severe inactivation of enzymes in high concentrations of lactic acid or in solvent-free systems, as it decreases the logP of the reaction medium (Pirozzi and Greco 2004). Polar solvents aid lactic acid solubilization at higher concentrations and seem to prevent enzyme inactivation because they show an acid-suppressive effect due to their basicity (Hasegawa et al. 2008). However, esterification of glycolic acid has been favored in apolar hexane producing high yield of glycolate ester (91 % after 24 h) (Torres and Otero 1999). Limitation of lactic acid self-polymerization has been achieved in hexane although the esterification with fatty acids resulted in lower yields (35 %) (Torres and Otero 2001). Transesterification between α-butyl glycoside and butyl lactate in a solvent-free system eliminating the butanol co-product under reduced pressure resulted in more than 95 % conversion and very high concentration of a less irritant product (170 g/L) in a single batch reaction (Bousquet et al. 1999).

### Kojic acid derivatives

Kojic acid (5-hydroxy-2-(hydroxymethyl)-4H-pyran-4-one) is an inexpensive water-soluble fungal secondary metabolite produced by *Aspergillus* and *Penicillium* species. It possesses valuable biological properties such as anti-oxidant, anti-microbial, and anti-inflammatory, while as an iron and copper chelator has the capacity to prevent photodamage, hyperpigmentation, and skin wrinkling. Its primary use in cosmetics is as a skin whitening agent but there are concerns regarding its skin compatibility, oil solubility, and storage stability even at ordinary temperatures. Additionally, there is evidence of toxicity, irritancy, and carcinogenicity (Lajis et al. 2012). The first attempts on the enzymatic modification of kojic acid focused on the synthesis of kojic acid glycosides using a sucrose phosphorylase from *Leuconostoc mesenteroides*, an α-amylase from *Bacillus subtilis* and an immobilized β-galactosidase from *Bacillus circulans* (Nishimura et al. 1994; Kitao and Serine 1994; Hassan et al. 1995). However, many lipophilic derivatives such as saturated fatty (C6-C18) acid esters and the unsaturated kojic acid monoricinolate and monooleate have been synthesized by commercial lipases (Liu and Shaw 1998; Lajis et al. 2013; Khamaruddin et al. 2008; El-Boulifi et al. 2014; Ashari et al. 2009). A phospholipase from *Streptomyces* sp. has synthesized phosphatidylkojic acid at 60 % yield from a dipalmitoylphosphatidyl residue (Takami et al. 1994). Kojic acid has two OH– groups, the primary at C-7 and the secondary one at C-5 which is essential to the radical scavenging and tyrosinase interference activity. Many derivatives have been synthesized by modifying the 5-OH group; nevertheless, CALB showed moderate yield (53 %) synthesizing a laurate product esterified at the primary C-7 (Kobayashi et al. 2001; Chen et al. 2002).

### Lipoic acid derivatives

α-Lipoic acid is a dithiol compound containing two sulfur atoms at the C-6 and C-8 carbons connected by a disulfide bond. It takes part in the anti-oxidant defense system of the cell through its ability to scavenge free radicals both in lipid and aqueous environments. This amphiphilicity constitutes it an ideal candidate for use in both oil- and water-based formulations. Moreover, it participates in the regeneration of anti-oxidants (i.e., vitamic C, vitamin E) and in the de novo synthesis of endogenous anti-oxidants (i.e., glutathione) and shows metal ion chelating activity, while it can repair oxidative damage in macromolecules (Papadopoulou et al. 2013). Other attractive properties include anti-inflammatory activity, aid in the treatment of diseases such as diabetes, atherosclerosis, cardiovascular, heavy-metal poisoning, radiation damage, cancer, Alzheimer’s, and AIDS (Maczurek et al. 2008). Synthesis of lipoic acid phenolic derivatives by CALB showed that a prior aromatic hydroxylation of the donor offered higher 2,2-diphenyl-1-picrylhydrazyl (DPPH) radical scavenging activity to the products. The hydroxytyrosol ester of lipoic acid showed similar anti-oxidant activity to α-tocopherol but higher than the commercial butylated hydroxytoluene (BHT) (Kaki et al. 2012). Lipoic acid is found in a racemic mixture, where the (R)-enantiomer is much more active than the (S)-enantiomer. Only lipases from *Candida rugosa* and *Aspergillus oryzae* (whole cell) have been reported to enable kinetic resolution of racemic α-lipoic acid (Yan et al. 2009; Fadnavis et al. 1998).

### Hydroquinone derivatives

Hydroquinone, a phenolic compound with two –OH groups at the para positions of the benzene ring, has been commercially used in cosmetics at concentrations <1 % as an anti-oxidant, fragrance, reducing agent, or polymerization inhibitor (Andersen et al. 2010). Its most promising use is as a skin whitening agent; however, it is prone to cause irritations and dermatitis (Kang et al. 2009). Its glycosylated derivative, arbutin, has attracted attention as a better tyrosinase inhibitor when compared to conventional agents as it inhibits melanogenesis without causing melanocytotoxicity (Sugimoto et al. 2005). It also plays an important role in scavenging free radicals, as an anti-inflammatory, and an anti-microbial agent (Lee and Kim 2012). Αrbutin has two isomers (α- and β-). The first is synthesized by chemical or enzymatic methods and shows higher efficiency and stability while the latter is extracted from natural sources such as bearberry, cranberry, blueberry, and pears (Seo et al. 2012a). α-Arbutin possesses a 10-fold stronger inhibitory effect on the activity of tyrosinase from human malignant melanoma cells compared to its anomer, whereas β-arbutin reduces tyrosinase activities from mushroom and mouse melanoma (Seo et al. 2012b). α-Arbutin shows extremely increased browning resistance to light irradiation compared to hydroquinone (Kitao and Sekine 1994). Lipases have been used for the acylation of β-arbutin with aromatic or fatty acids showing absolute regioselectivity at the 6′ position. This phenomenon can be attributed to the hypothesis that the primary OH– group of the sugar moiety is less hindered so it can enter more easily into the active site of the lipase and attach the acyl-enzyme intermediate. Studies on immobilized lipase from *Penicillium expansum* showed that the elongation of the donor chain length (C2–C8) results in higher initial yields perhaps due to stronger interactions with the hydrophobic acyl binding site of the enzyme. Branched-chain acyl donors affect negatively the initial rate due to steric strain while the presence of a conjugated C–C double bond adjacent to the carbonyl moiety decreases the rate substantially (Yang et al. 2010a). The radical scavenging activity of acyl (C6–C18) arbutin is independent of the chain length (Nagai et al. 2009). Fatty acid derivatives of arbutin show higher anti-melanogenesis and anti-oxidant activity than arbutin which could be allied to the improved membrane penetration, due to increased lipophilicity (Watanabe et al. 2009). Synthesized by CALB, arbutin ferulate was found to have 19 % higher activity against the 2,2′-azino-bis(3-ethylbenzothiazoline-6-sulphonic acid (ABTS) free radical than ferulic acid and be 10 % more efficient towards low-density lipoprotein (LDL), showing higher anti-oxidant than Trolox, a well-known analog of vitamin E and commercial anti-oxidant (Chigorimbo-Murefu et al. 2009).

### Vitamin derivatives

L-Ascorbic acid (vitamin C) is a potent water-soluble natural anti-oxidant that has been used in cosmetics as a preservative, pH adjuster, or/and an active compound. It has been proved that ascorbates promote collagen synthesis in human skin fibroblasts in vitro up to eightfold capacity, while they show photoprotective activity against UVA and UVB irradiation and have wound healing properties (Murad et al. 1981). Drawbacks as instability, poor liposolubility, and low skin penetrability have led to modifications. Common saturated fatty acid derivatives, as ascorbyl palmitate and ascorbyl stearate, have been synthesized showing that there is no negative effect on the radical scavenging activity by introducing a saturated group at the C-6 position of ascorbic acid (Watanabe et al. 2003). Enzymatic synthesis of ascorbyl palmitate is focused on the esterification of palmitic acid with a vast use of CALB in organic solvents or ionic liquids. Other commercial lipases have been employed offering varying yields (6–97 %) (Gulati et al. 1999; Costa et al. 2014; Park et al. 2003; Hsieh et al. 2006, Bradoo et al. 1999). However, saturated fatty acid esters still show moderate solubility in oils. Further improvement can be done by introducing a double bond in the fatty acid, resulting in superior products in terms of solubility and radical scavenging capacity. For instance, oleic acid is readily available and inexpensive (Viklund et al. 2003). There are reports on esterification of olive oil, palm oil, or lard offering a simple, direct, and natural route for synthesis (Moreno-Perez et al. 2013; Zhao et al. 2014; Burham et al. 2009). Derivatization of L-ascorbic acid requires mild conditions to prevent oxidation of both acid and its esters and high regioselectivity for the 6-*O*-position which is achieved by lipases (Zhang et al. 2012). However, the demand of polar solvents for enhancing substrate solubility tends to be deleterious for their stability. Coating is an effective way to protect immobilized lipases from denaturation reducing the interactions with the solvent (Moreno-Perez et al. 2013). The use of vinyl ester donors increases the reaction rate, but implies the release of fatty acids from oils and their further activation. For instance, CALB offered 100 % conversion of vinyl palmitate in *t*-butanol (Reyes-Duarte et al. 2011). When methyl esters are used, the by-product methanol is insoluble in oils, gets adsorbed onto the surface of the immobilized lipase, and leads to negative effects on enzyme activity and operational stability.

Vitamin E is a general term for a group of amphiphilic lipids, comprising of four tocopherols, having a saturated phytyl side chain attached to the chromanol core and four tocotrienols having an attached unsaturated isoprenoid side chain. The analogs differ with each other by the presence and placement of methyl groups around the aromatic ring. In nature, vitamin E occurs only in the *RRR*-form, while *RRR-*α-tocopherol is the most bioactive. Synthetic vitamin E (α-tocopherol) is a racemic mixture of eight stereoisomers in equal amounts (*all-rac*-α), of which not all are as bioactive as the natural form (Torres et al. 2008a). Vitamin E is non-irritant to the eyes and skin, has high anti-oxidant activity with anti-aging and anti-tumor potential, inhibits the UVB-induced lipid peroxidation, and shows skin-improving properties with anti-inflammatory and beneficial effect on the skin barrier function (Zondlo Fiume 2002). However, it is readily destabilized by light and oxygen. Non-enantioselective acetylation of vitamin E at the C-6 carbon has been performed only by CALB among other tested enzymes which can be explained by studies that show that the acceptor binding site is deeper in lipase B (Torres et al. 2008b; Pleiss et al. 1998). δ-Tocopherol gave higher rates due to its lower methylation degree, while competitive acetylation experiments indicated that there is steric hindrance caused by the aliphatic chain and not the chromanol ring. Vitamin E succinate has been synthesized by modified CALB yielding 94 % and by a lipase from *C. rugosa* with moderate yields (Yin et al. 2011; Jiang et al. 2013). Synthesized at lower yields (25.2 %) by CALB, novel vitamin E ferulate inhibits melanogenesis in human melanoma cells, being an attractive candidate as a skin-whitening agent (Xin et al. 2011).

Vitamin A includes a group of unsaturated compounds, i.e., retinol, retinoic acid, and retinaldehyde, which are widely used in cosmetic and skin care products because of their anti-oxidant, anti-aging, and skin-whitening properties. Retinol is the most active form of vitamin A; however, retinoids are readily oxidized in air and inactivated by UV light while they are water-insoluble and skin-irritating (Maugard and Legoy 2000). The most common modification of retinol is retinyl palmitate, which is stable, slightly irritating, and not sensitizing at concentrations between 0.1 and 1 % (CIR 1987). It has been synthesized by the esterification of palmitic acid using CALB, a lipase from *Candida* sp. and a modified lipase from *Pseudomonas fluorescens* (Ajima et al. 1986; Yin et al. 2006; Liu et al. 2012). Other vitamin A modifications include saturated fatty acid esters, oleate, lactate, and succinate/methylsuccinate derivatives catalyzed by CALB or *Rhizomucor miehei* lipase (Maugard and Legoy 2000; Liu et al. 2012). Rejasse et al. (2003) proposed a vitamin A inter-esterification reaction using CALB. The first step included esterification of adipic acid with retinol in *t*-amyl alcohol, while after 24 h, a polyol was added resulting in products with varying yields (22–80 %).

### Flavonoid derivatives

Aglycon and glycosylated flavonoids are natural compounds of plant origin that have aroused interest for their anti-viral, anti-allergic, anti-inflammatory, anti-oxidant activities, and the protection against cardiovascular diseases and cancer (Salas et al. 2011). Their basic structure is derived from 2-phenylbenzo-γ-pyran, where the original skeleton is substituted with numerous OH– groups that result in a considerably hydrophilic nature. The effect of acyl donors on esculin and rutin modification by CALB has been studied in microreactors offering conversion rates higher than 9.5 10^−2^ mmol L^−1^ h^−1^(Ardhaoui et al. 2004a). Naringin esterification in a continuous flow microreactor offered more than 85 % conversion to 6-O″-monoesters. Regioselective acylation in microreactors offers mild reaction conditions, short reaction times, and high yields (Luo et al. 2013). Vinyl esters of saturated fatty acids are more reactive giving approximately a threefold increase in the conversion of naringin (Mellou et al. 2005). The enzymatic acylation of two isolated chrysoeriol glucosides by CALB resulted in products with higher anti-oxidant and anti-microbial activity against Gram-negative and Gram-positive bacteria that can be attributed to the increased interaction of the hydrophobic chain with the cell membrane due to modified lipophilicity. Irilone, chrysin, and dihydromyricetin acetate have been synthesized by *Pseudomonas* (syn *Burkholderia*) *cepacia* lipases and an immobilized lipase from *P. expansum* (Nazir et al. 2009; Chebil et al. 2007; Li et al. 2015). Orientin, vitexin, salicin fatty acid esters, and helicin butyrate have been synthesized by CALB (Liu et al. 2015; Katsoura et al. 2007). Silibyn, which occurs in nature as an equimolar mixture of two diastereoisomers (A and B) with different biological activities, has been acylated by CALB at the C-23 position producing new anti-viral and anti-tumor compounds (Gazak et al. 2010). Modification (e.g., methylation) of the C-7 OH which bears a pro-oxidant potential significantly improves the anti-radical activity of silybin.

The nature of flavonoid and the origin of lipase are crucial for product formation. Generally, flavonoids with a primary OH– group on the glycosyl moiety as naringin are more reactive than those with secondary OH– groups only, as rutin. Chebil et al. (2007) showed that isoquercetrin, the glycosylated form of quercetin, could be acylated by both CALB and *P. cepacia* lipase (PSL) although only the latter could acylate quercetin. In the absence of the 4′-OH group of quercetin (hesperetin), PSL showed preference for the 7-OH group, followed by the 3′-OH group which can be explained by steric hindrance from the C-4′ methoxy group. Chrysin was acylated only at the 7-OH group since the 5-OH group is not reactive when a 4-oxo group is present in the structure of the flavonoid. Molecular modeling regarding the regioselectivity of CALB showed that the aglycon part of both rutin and isoquercitrin was localized at the entrance of the enzyme’s binding pocket stabilized by hydrogen bond and hydrophobic interactions (de Oliveira et al. 2009). The sugar part was placed close to the pocket bottom. Only the primary 6′-OH group of isoquercitrin glucose and the secondary 4″-OH group of rutin rhamnose were expected to be acetylated as they were the only ones to stabilize simultaneously near the catalytic histidine and the acetate bound to the catalytic serine. CALB synthesized monoesters on the primary OH of glucose moiety of esculin and on the secondary 4″′-OH of the rhamnose residue of rutin (Ardhaoui et al. 2004b). Acylation of quercetin was not achieved as the 4′-OH is conjugated with the C-4 carbonyl group favoring a planar formation of the molecule, which may not be suitable for the catalytic site of the enzyme (Nazir et al. 2009).

### Hydroxycinnamic acid derivatives

Hydroxycinnamic acids (HCAs; ferulic, FA; *p*-coumaric, p-CA; caffeic, CA; sinapic, SA) are a class of phenylpropanoids known as more active anti-oxidants than hydroxybenzoic acids due to the presence of the C=COOH group (Widjaja et al. 2008). They are ubiquitous in nature as a component of arabinoxylans in plant cell walls offering linkage with lignin while they present broad spectrum of biological activities including anti-bacterial, anti-viral, anti-inflammatory, anti-carcinogenic, anti-HIV, and anti-tumor effects (Tan and Shahidi 2012). However, their solubility is poor in hydrophilic and lipophilic media. Among many natural photoprotective agents, feruloylated lipids have gained attention due to their strong anti-oxidant, skin-whitening, anti-wrinkling, and UV absorptive ability (Radzi et al. 2014). FA is believed to suppress melanin generation by antagonizing tyrosine because its structure is similar to tyrosine (Chandel et al. 2011). Enzymatic synthesis of green sunscreens offers stability and selectivity in contrast with chemical synthesis that is limited due to heat sensitivity and oxidation susceptibility of FA in alkaline media. A two-step synthesis of feruloylated diacylglycerols using CALB has been proposed by Sun et al. (2007) including the transesterification of ethyl ferulate with glycerol and the subsequent transesterification of glyceryl ferulate with oleic acid offering high yield of products (up to 96 %). Esterification of FA to glyceryl ferulate by CALB has been performed in a continuous reactor reaching 80 % conversion and productivity of 430 kg/m^3^/reactor day (Matsuo et al. 2008). Biocatalysis under vacuum-rotary evaporation contributes to increased conversion by eliminating external mass transfer resistance, effective interaction among different phases of enzymatic reaction, minimizing the negative effects of by-product ethanol (when ethyl ferulate is used as donor) on lipase activity (Xin et al. 2009). 1,3-Diferuloyl-*sn*-glycerol has been synthesized by CALB in a pilot plant scale bed reactor as by-product of the transesterification of ethyl ferulate with soybean oil (Compton and Laszlo 2009). One hundred twenty kilograms of diferuloyl glycerol by-product could be isolated annually. Enzymatic esterification of olive, flaxseed, and fish oil offers low cost and greener configurations to the process (Ciftci and Saldana 2012; Yang et al. 2012; Radzi et al. 2014). Transesterification of ethyl ferulate with castor oil, catalyzed by CALB, yielded 62.6 % lipophilic and 37.3 % hydrophilic products (Sun et al. 2014). Esterification of FA with fatty (C2–C8) alcohols improves the anti-oxidant capacity towards the oxidation of HDL, LDL, and total serum. Probably, the lipophilic properties of anti-oxidants affect their incorporation into the lipid part of lipoproteins reaching the site of lipoperoxidation, accounting for the increased anti-oxidant activity (Jakovetic et al. 2013).

Transesterification of methyl caffeate to propyl caffeate by CALB was performed in a continuous flow packed bed microreactor offering 99.5 % yield. The calculated kinetic constant *K*_*m*_ was 16 times lower than than of a batch reactor (Wang et al. 2013). Caffeic acid phenethyl ester (CAPE) is a flavonoid-like compound and one of the major components of honeybee propolis possessing anti-inflammatory, anti-carcinogenic, and neuroprotective properties (Widjaja et al. 2008). High yield CAPE synthesis has been performed by CALB in a packed bed reactor, using ultrasound treatment or in one-pot reactions using organic solvents or ionic liquids (Chen et al. 2010, 2011a; Ha et al. 2012; Wang et al. 2014). One-pot synthesis of a CAPE analog, 3-cyclohexyl caffeate, has been performed by esterification of caffeoylquinic acids derived from coffee beans with methanol using a chlorogenate hydrolase followed by the transesterification of methyl caffeate with 3-cyclohecylpropyl caffeate using CALB in [Bmim][NTf_2_] (Kurata et al. 2011). Synthesized by a *C. rugosa* lipase, phytosteryl caffeate showed twofold increase in oxygen radical absorbance capacity (ORAC) comparing to the parent vinyl HCA, while phytosteryl ferulate showed a 10-fold increased anti-oxidant activity compared to Trolox and a twofold increase comparing to vinyl ferulate (Tan and Shahidi 2011, 2012). Chigorimbo-Murefu et al. (2009) synthesized arbutin and hydroxyl steroid esters of FA in *t*-methyl-ethyl ether showing higher anti-oxidant activity than Trolox and their starting hydroxycinnamate. Arbutin ferulate possessed 19 % higher anti-radical activity against ABTS free radical than FA and inhibited 10 % more efficiently LDL oxidation than its precursors.

Although FAEs are less stable in organic media and low water content than lipases, they show higher substrate specificity (Zeuner et al. 2011). Some examples of FAE-catalyzed reactions are presented in Table 2. In 2001, Giuliani et al. succeeded for the first time the synthesis of 1-pentyl-ferulate using a FAE from *Aspergillus niger* in a water-in-oil microemulsions. Since then, novel FAEs from filamentous fungi such as *Fusarium oxysporum*, *Myceliophthora thermophila* (syn *Sporotrichum thermophile*), and *Talaromyces stipitatus* have been employed in detergentless microemulsions for the transesterification of methyl donors to alkyl hydroxycinnamates, feruloylated-arabino-oligosaccharides showing regioselectivity for the primary hydroxyl group of the non-reducing arabinofuranose ring and other sugar ferulates (Topakas et al. 2003a; Vafiadi et al. 2005, 2006, 2007b, 2008a). Although esterification with fatty alcohols generally results in more lipophilic products, the glyceryl esters of HCAs have been proved more hydrophilic than their donors. Fed-batch esterification of FA with diglycerin was performed by a FAE from *A. niger* under reduced pressure yielding 69 % feruloyl and 21 % diferuloyl diglycerols (Kikugawa et al. 2012). The major product (FA-DG1) showed highest water solubility while all products maintained their radical scavenging activity against DPPH and their UV absorption properties. Diferuloyl diglycerols showed a twofold increase of anti-oxidant activity comparing to feruloyl diglycerols and FA. Esterification of SA and *p*-CA with glycerol yielded 70 % glycerol sinapate and 60 % glycerol-*p*-coumarate, respectively, with indication of the formation of minor dicinnamoyl glycerol esters (Tsuchiyama et al. 2007). The ability of glycerol sinapate to scavenge DPHH radicals was higher than BHT while it maintained its UV absorption properties.

**Table 2:** Ferulic acid esterase-catalyzed reactions

Product	Donor	Acceptor	Enzyme	Solvent system	Yield (time)	T (°C)	Reference
1-Pentyl ferulate	Ferulic acid	1-Pentanol	FAEA	CTAB: hexane: pentanol: buffer	60 % (n.q.)	40	Giuliani et al. 2001
1-Butyl ferulate	Methyl ferulate	1-Butanol	CLEAs immobilized Ultraflo L	Hexane: 1-butanol: buffer	97 % (144 h)	37	Vafiadi et al. 2008a
1-Butyl sinapate	Methyl sinapate	1-Butanol	AnFaeA	Hexane: 1-butanol: buffer	78 % (120 h)	35	Vafiadi et al. 2008b
2-Butyl sinapate	Methyl sinapate	2-Butanol	AnFaeA	Hexane: 2-butanol: buffer	9 % (120 h)	37	Vafiadi et al. 2008a
1-Butyl caffeate	Methyl caffeate	1-Butanol	StFae-A	Hexane: 1-butanol: buffer	Up to 25 % (144 h)	35	Topakas et al. 2004
1-Butyl-*p*-coumarate	Methyl *p*-coumarate	1-Butanol	FoFae-I	Hexane: 1-butanol: buffer	Up to 70 % (144 h)	35	Topakas et al. 2003a
1-Propyl-*p*-hydroxyphenylacetate	*p*-Hydroxyphenylacetic acid	1-Propanol	FoFae-II	Hexane: 1-propanol: buffer	75 % (224 h)	30	Topakas et al. 2003b
1-Propyl-*p*-hydroxylphenylpropionate	*p*-Hydroxylphenylpropionic acid				70 % (224 h)		
Glycerol sinapate	Sinapic acid	Glycerol	AnFaeA	[C_5_OHmim][PF_6_]: buffer	76.7 % (24 h)	50	Vafiadi et al. 2009
	Methyl sinapate				Up to 7 % (120 h)		
Glycerol ferulate	Ferulic acid	Glycerol	FAE-PL	Glycerol: DMSO: buffer	81 % (n.q.)	50	Tsuchiyama et al. 2006
Diglycerol ferulates (mixture of isomers)	Ferulic acid	Diglycerin S	FAE-PL	Diglycerin S: DMSO: buffer	95 % (12 h)	50	Kikugawa et al. 2012
Glycerol *p*-coumarate	*p*-Coumaric acid	Glycerol	FAE-PL	Glycerol: DMSO: buffer	~60 % (72 h)	50	Tsuchiyama et al. 2007
*L*-Arabinose ferulate	Methyl ferulate	*L*-Arabinose	StFae-C	Hexane: *t*-butanol: buffer	Up to 50 % (120 h)	35	Vafiadi et al. 2005
	Ethyl ferulate				6.3 % (−)		
*D*-Arabinose ferulate	Methyl ferulate	*D*-Arabinose		Hexane: *t*-butanol: buffer	45 % (−)	35	Vafiadi et al. 2007a
	Ferulic acid	*D*-Arabinose	Multifect P3000	Hexane: 1-butanol:buffer	36.7 % (144 h)	35	Couto et al. 2010
*D*-Galactose ferulate	Ferulic acid	*D*-Galactose	Depol 670		61.5 % (144 h)		
*D*-Xylose ferulate	Ferulic acid	D-Xylose		Hexane: 2-butanone:buffer	37.3 % (144 h)		
Feruloyl raffinose	Ferulic acid	Raffinose	Depol 740L	Hexane: 2-butanone:buffer	11.9 % (7 days)	35	Couto et al. 2011
Feruloyl galactobiose	Ferulic acid	Galactobiose		Hexane: 1,4-dioxane:buffer	26.8 % (144 h)		
Feruloyl xylobiose	Ferulic acid	Xylobiose		Hexane: 2-butanone:buffer	9.4 % (144 h)		
Feruloyl arabinodiose	Ferulic acid	Arabinodiose			7.9 % (144 h)		
Feruloyl sucrose	Ferulic acid	Sucrose			13.2 % (n.q.)		
Feruloyl FOS	Ferulic acid	FOS			9.6 % (n.q.)		

FAEA: FAE from *Aspergillus niger*; Ultraflo L, Depol 740L: multi-enzymatic preparation from *Humicola insolens*; AnFaeA: type A FAE from *A. niger*; StFae-A, StFae-C: FAE from *Sporotrichum thermophile* ATCC 34628; FoFae-I, FoFae-II: FAE from *Fusarium oxysporum*; FAE-PL: FAE from *A. niger* purified from the commercial preparation Pectinase PL“Amano”; Multifect P3000: multi-enzymatic preparation from *Bacillus amyloliquefaciens*; Depol 670: multi-enzymatic preparation from *Trichoderma reesei*

### Galloyl derivatives

Tannins, natural occurring polyphenols that can be found in pine and spruce bark, vegetables, and fruits, are categorized into hydrolysable, condensed, and complex. The simplest hydrolysable tannins, commonly named gallotannins, consist of gallic acid molecules esterified to a core polyol. The biocatalytic synthesis of gallic acid esters is performed mainly by tannases and may follow different routes: (1) hydrolysis of tannic acid into gallic acid and further esterification to galloyl esters, (2) direct esterification of tannic acid into a galloyl ester, or (3) transesterification of galloyl esters into another ester. Examples of tannase-based reactions are presented in Table 3. A well-known synthetic galloyl ester widely used in skin cleaning/care products, make up, sunscreen, and tanning products is propyl gallate. Its biological activities are not limited to the free-radical scavenging ability as it exhibits anti-microbial, anti-nociceptive activity, ultraviolet (UV) radiation protection, anti-cariogenesis, anti-mutagenesis, and anti-carcinogenesis effects. However, in cosmetic formulations, its concentration is low (up to 0.1 %) due to indications for skin irritation or sensitization (CIR 2007). Applications of propyl gallate expand into the food, pharmaceutical, adhesive, lubricant, and biodiesel industry where it has been used as an anti-oxidant additive, for more than 20 years (Zhang 2015).

**Table 3:** Tannase-catalyzed reactions

Product	Donor	Acceptor	Enzyme	Solvent system	Yield (time)	T (°C)	Reference
Methyl gallate	Gallic acid	Methanol	Tannase from *Aspergillus niger*	Hexane	90.7 % (8 h)	50	Sharma and Saxena 2012
Propyl gallate		1-Propanol			94.8 % (8 h)		
	Tannic acid		CNBr-agarose immobilized tannase from *Emericela ridulans*	Buffer	88 % (48 h)	60–75	Goncalves et al. 2013
	Methyl gallate		CNBr-agarose immobilized tannase from *Lactobacillus plantarum*	Buffer	55 % (−)	25	Fernandez-Lorente et al. 2011
C1–C12 acyl gallates	Gallic acid	C1–C12 fatty alcohols	Tannase from *Aspergillus niger* immobilized on alkylaminosilanized porous silica	Solvent-free	10–95 % (18–48 h)	RT	Weetall 1985
C3–C5 diol gallates (strong indication of more than one form of ester)		Diols			50–80 % (24 h)		
Catechin gallate	Gallic acid	Catechin	Tannase from *Aspergillus niger* immobilized on Eupergit C	[ΒMIM][MEESO_4_]:buffer	1.3 % (20 h)	RT	Raab et al. 2007
Epicatechin gallate		Epicatechin			5.4 % (20 h)		
Epigallocatechin gallate		Epigallocatechin			3.1 % (20 h)		

The majority of tannases used for the synthesis of propyl gallate are carrier-bound or modified. A mycelium-bound tannase from *A. niger* esterified gallic acid at 65 % yield (Yu et al. 2007), whereas its microencapsulation by a chitosan-alginate complex showed more moderate results (Yu and Li 2005). Mycelia could protect the enzyme from the harshness of organic solvents as an immobilization matrix does and offer avoidance of costly and time-consuming purifications. Tannases from *Aspergillus* species, *Lactobacillus plantarum*, and *Emericella nidulans* immobilized on different carriers, catalyzed the esterification of tannic acid in organic and aqueous media offering high yields (43–88 %) (Fernandez-Lorente et al. 2011; Prasad et al. 2011; Nie et al. 2012a; Goncalves et al. 2013). A bioimprinted commercial tannase esterified tannic acid with propanol resulting in 50 % yield increase compared to the non-imprinted enzyme. Bioimprinting locks the enzyme into a favorable conformation for catalysis during lyophilization through the addition of the targeted substrate prior to freezing. Moreover, the ligand may impede the formation of inactive “microconformations” in the active site (Aithal and Belur 2013). Bioimprinting methods can activate tannase remarkably offering a 100-fold increase of conversion (Nie et al. 2012b). Techniques such as pH tuning, interfacial activation, and cryogenic protection have been applied (Nie et al. 2012a, 2014). Free tannases from *Aspergillus* species, *Penicillium variable*, and *Bacillus massiliensis* (whole-cell) have synthesized propyl gallate in organic solvents (Yu and Li 2008; Sharma and Gupta 2003; Sharma and Saxena 2012; Beena et al. 2011). Regarding other galloyl esters, Toth and Hensler (1952) reported the synthesis of methyl and ethyl esters but not the phenyl ester of gallic acid in the presence of tannase dissolved in buffer, revealing for the first time the ability of soluble tannases to esterify. Gallic acid esters were synthesized by an immobilized tannase from *A. niger* performing esterification of gallic acid with alcohols (C1–C12) and with several diols. This system proved that the enzyme had broad specificity for alcohols but absolute specificity for the acid moiety (Weetall 1985).

Representing proanthocyanidin monomers, green tea catechins mainly comprising of epicatechins (ECs), epigallocatechins (EGCs), epicatechin gallate (ECG), and epigallocatechin gallate (EGCG) have gained attention for their strong anti-oxidant and cardiovascular protective activity. Green tea is considered a useful agent for promoting skin regeneration or treatment for psoriasis, rosacea, and actinic keratosis and repairs UV-damaged skin in vivo, which leads to the improvement of wrinkles (Hong et al. 2012). EGCG is an anti-inflammatory and anti-irritant anti-oxidant, which is responsible for health benefits like the stimulation of collagen production while reducing oxidative damage within the skin. EGCG vehiculated in cosmetic formulations presents good skin penetration and retention favoring its skin effects (dal Belo et al. 2009). Among epicatechin derivatives, EGC is the most effective photoprotector, following in a descending order by EGCG, EC, and ECG (Hong et al. 2013). However, it is present in natural green tea preparations in low amounts compared to EGCG, which is the most abundant catechin in green tea (Cao and Ito 2004). Low-yield galloylation of epicatechins has been achieved by an immobilized commercial tannase from *A. niger* in ionic liquids (Raab et al. 2007). It is evident that tannases could be proved to be a powerful biocatalyst in order to modify the active constituents of green tea and synthesize tailor-made compounds with preferred biological activities for use in different cosmeceutical products. High yield acetylation of catechin and ECGG has been reported using commercial lipases from *R. miehei* and *P. cepacia* (Lambusta et al. 1993; Zhu et al. 2014).

## Proteases

Besides catalyzing the cleavage of peptide bonds for the production of peptide cosmeceuticals, proteases (EC 3.4) find application in transesterification reactions in organic solvents, lowering the cost of ester production and increasing reaction specificity. Enzymes from different sources display different features; for example, contrary to serine proteases, thermolysin (a metallo-protease from *Bacillus thermoproteolyticus*) is not generally used in transesterifications (Pedersen et al. 2002). Studies have proved that the S1 pocket of thermolysin active site can bind medium and large hydrophobic amino acids, suggesting that the vinyl group can bind as well, allowing the possibility of using thermolysin for the synthesis of sugar esters. For these reasons, the use of proteases for ester production in the cosmetic field is of great interest and potential (Fornbacke and Clarsund 2013). The main compounds synthesized by proteases are summarized in Table 4.

**Table 4:** Protease-catalyzed reactions

Product	Donor	Acceptor	Enzyme	Solvent system	Yield (time)	T (°C)	Reference
7-*O*-Vinyl adipoyl kojic acid	Kojic acid	Divinyl adipate	Bioprase from *Bacillus subtilis*	Dimethylformamide	25 % (7 days)	30	Raku and Tokiwa 2003
7-*O*-Hexanoyl/octanoyl/decanoyl kojic acid		Vinyl hexanoate/octanoate/decanoate			13–27 % (7 days)		
6-*O*-Undecylenoyl p-hydroxyphenyl β-D-glucopyranoside	Arbutin	Undecylenic acid vinyl ester	Bioprase from *Bacillus subtilis*	Dimethylformamide	62 % (7 days)	40	Tokiwa et al. 2007b
3″-*O*-Vinylsuccinyl or vinylsebacoyl-rutin	Rutin	Divinyl succinate/sebacate	Subtilisin from *Bacillus subtilis*	Pyridine	12.8/19.8 % (4 days)	50	Xiao et al. 2005
Vinylsuccinyl/vinylglutaryl/vinyladipoyl/dinylnonanedioyl/vinylsebacoyl/vinyltridecanedioyl-troxerutin	Troxerutin	Divinyl succinate/glutarate/adipate/nonanedioate/sebacate/decanedioate	Subtilisin from *Bacillus subtilis*(enzyme pre-irradiated)	Pyridine	10.6–33.10 % (4 h)	50	Xiao et al. 2011
2-*O*-Lauroyl-sucrose	Sucrose	Vinyl laurate	Alkaline protease from *Bacillus pseudofirmus*	Dimethylformamide:pyridine	50–60 % (24 h)	45	Pedersen et al. 2003
6-O-Vinyladipoyl-D-glucose/-D-mannose/-D-galactose/-methyl D-galactoside	D-Glucose/D-mannose/D-galactose/α-methyl D-galactoside	Divinyl adipate	Alkaline protease from *Streptomyces* sp.	Dimethylformamide	49–74 % (7 days)	35	Kitagawa et al. 1999

As a typical flavonoid glycoside with anti-oxidant activity, rutin has been enzymatically esterified with different acyl donors to enhance its solubility and stability in lipophilic media. The regioselective transesterification of rutin with divinyl carboxylates in pyridine was performed at 50 °C for 4 days by an alkaline protease from *B. subtilis* (Xiao et al. 2005). Only 3″-*O*-substituted rutin ester was obtained in these conditions showing that regioselective acylation can be controlled by regulation of solvents and enzymes. Pre-irradiated alkaline protease from *B. subtilis* increased transesterification of troxerutin with divinyl dicarboxylates by 31 % in pyridine using an ultrasound bath (150 W and 80 kHz) (Xiao et al. 2011). Ultrasonic treatment is an environmentally benign method based on the cavitation phenomenon, which causes the formation, expansion, and collapse of cavities generating high temperatures and pressures of the neighboring surroundings (Khan and Rathod 2015). Thus, cavitation can accelerate enzymatic reactions maintaining ambient conditions of the overall environment. Ultrasonic treatment represents an efficient route of performing transterification in order to modify solubility of anti-oxidant molecules.

Arbutin derivative with undecylenic acid located at its sugar moiety has been enzymatically synthesized using an alkaline protease from *B. subtilis* in a mixture of dimethylformamide and water (95:5) (Tokiwa et al. 2007a). The produced arbutin undecylenic acid ester showed to inhibit the activity of tyrosinase from mushroom; in addition, the arbutin ester seemed to have high dermal absortion and did not show the peculiar smell of undecylenic acid which commonly prevents its application in cosmetics. Further studies have proven that after 6 days of incubation of B16 melanoma cells with arbutin undecylenic ester, melanin production levels were decreased to approximately 30 % compared with that of the control cells (Tokiwa et al. 2007b). Alkaline protease from *B. subtilis* was also applied in regioselective esterification of the hydroxyl group at C-7 position of kojic acid to produce diverse lipophilic kojic acid esters in dimethylformamide (Raku and Tokiwa 2003). Kojic acid esters were effective as scavengers against DPPH radical, and they are expected to prevent oxidational stress in vivo. Moreover, their biodegradability exceeded 60 %, allowing their application in cosmetics for the production of eco-friendly and oil-based product products.

## Transferases

A broad variety of bioactive glycosides has been synthesized using glycosyltransferases (GTFs; EC 2.4); enzymes that catalyze the transfer of sugar moieties from an activated donor to specific acceptors forming glycosidic bonds. Novel EGCG mono- and di-glycosides with increased UV irradiation stability, browning resistance, and water solubility regardless of the position or linkage of the glycosylation have been synthesized by transferases from *L. mesenteroides* (Kitao et al. 1995; Moon et al. 2006a). EC mono-, di-, and tri-glycosides have been synthesized by a β-cyclodextrin glucosyltransferase from *Paenibacillus* sp. while various catechin derivatives by amylosucrases from *Deinococcus geothermalis*, *Streptococcus sobrinus*, a cyclodextrin glucanotransferase from *Bacillus macerans* and an enzyme with glycosyl transfer activity from *Xanthomonas campestris* (Aramsangtienchai et al. 2011; Cho et al. 2011; Nakahara et al. 1995; Funayama et al. 1993; Sato et al. 2000). Transferase-based modification of hydroquinone has been focused on its glycosylation or the production of arbutin (α- and β-) glycosides. A two-step synthesis of α-arbutin including prior treatment of α-cyclodextrin with an amyloglucosidase from *A. niger* and subsequent transfer reaction using a commercial cyclodextrin glucanotransferase from *Thermoanaerobacter* sp. has been reported (Mathew and Adlercreutz 2013). Before treatment, hydroquinone was glycosylated with up to 7 glucose units while after treatment, α-arbutin was an absolute product. Results on the synthesis of arbutin glycosides show that the α-glucosidic linkage plays an important role in the inhibitory effect on human tyrosinase (Sugimoto et al. 2005).

2-*O*-α-D-glycopyranosyl L-ascorbic acid has been synthesized by a cyclomaltodextrin glucanotransferase from *Bacillus stearothermophilus* and a sucrose phosphorylase from *Bifidobacterium longum* (Aga et al. 1991; Kwon et al. 2007). The first transglycosylation of CA was performed by a sucrose phosphorylase from *B. longum* in aqueous CO_2_ supercritical media resulting in the formation of caffeic mono- and di-glycosides (Shin et al. 2009). Ampelopsin is a flavonoid with numerous activities such as anti-inflammatory, anti-microbial, anti-oxidant, anti-hypertension, hepatoprotective, anti-carcinogenic, anti-viral, and skin-whitening effects. A dextransucrase from *L. mesenteroides* synthesized a mixture of novel ampelopsin glycosides with up to 5 attached glycoside units. The primary product, ampelopsin-4′-*O*-α-D-glucopyranoside, reached an optimal yield of 34 g/L while it showed an 89-fold increase in water solubility, 14.5-fold increase in browning resistance comparing to ampelopsin, and 10-fold higher tyrosinase inhibition comparing to β-arbutin. Browning resistance was similar to ECGC glycosides and anti-oxidant activity superior to ampelopsin (Woo et al. 2012). Another major flavonoid found in plants, astragalin, was modified by a dextransucrase from *L. mesenteroides* giving products with increased inhibitory effects on matrix metalloproteinase-1 expression, anti-oxidant effect, and melanin inhibition (Kim et al. 2012). Quercetin glycosides were synthesized by a glucansucrase from *L. mesenteroides* showing increased water solubility, slower scavenging activity, and no improvement in tyrosinase inhibition (Moon et al. 2007b). Three main benzoyl and two main kojic acid glycosides were synthesized by a sucrose phosphorylase from *Streptococcus mutans* and *L. mesenteroides*, respectively (Sugimoto et al. 2007; Kitao and Serine 1994). Examples of transferase catalyzed reactions are presented in Table 5.

**Table 5:** Transferase catalyzed reactions

Product	Donor	Acceptor	Enzyme	Solvent system	Yield (time)	T (°C)	Reference
EGCG glycosides (EGCG-G1, EGCG-G2A, EGCG-G2B)	Sucrose	EGCG	Glucansucrase from *Leuconostoc mesenteroides*	Buffer	62.2 % (6.5 h)	28	Moon et al. 2006a
EC glycosides (EC3A, EC3B, EC3C, EC4A)	β-Cyclodextrin	(−)-Epicatechin	β-Cyclodextrin transferase from *Paenibacillus* sp.	Βuffer	18.1 % (24 h)	50	Aramsangtienchai et al. 2011
Catechin 3′-O-α-D-glucopyranoside	Maltose	(+)-Catechin	Glycosyltransferase from *Streptococcus sobrinus*	Buffer	13.7 % (24 h)	45	Nakahara et al. 1995
Catechin 3′-O-α-D-glucopyranoside (main product)	Starch		Cyclodextrin glucanotransferase from *Bacillus macerans*		18.3 % (40 h)	40	Funayama et al. 1993
Catechin 3′-O-α-D-glucopyranoside	Maltose		Enzyme with transfer activity from *Xanthomonas campestris* WU-9701		57.1 % (36 h)	45	Sato et al. 2000
Hydroquinone fructoside	Sucrose	Hydroquinone	Levansucrase from *Leuconostoc mesenteroides*	Buffer	14 % (6 h)	28	Kang et al. 2009
β-Αrbutin-α-G1/β-arbutin-α-G2	Sucrose	β-Arbutin	Glucansucrase from *Leuconostoc mesenteroides* B-1299B	Buffer	27.1 % (10 h)	28	Moon et al. 2007a
	Starch	β-Arbutin	Cyclomaltodextrin glucanotransferase from *Bacillus macerans*	Buffer	70 % (16 h)	40	Sugimoto et al. 2003
α-Αrbutin-α-G1/β-arbutin-α-G2		α-Arbutin		Buffer	70 % (16 h)	40	Sugimoto et al. 2005
α-Arbutin	Sucrose	Hydroquinone	Amylosucrase from *Deinococcus geothermalis*	Buffer	90 % (24 h)	35	Seo et al. 2012a
α-Arbutin (in a mixture of products, G2–G7)	α-Cyclodextrin		Cyclodextrin glycosyltranferase from *Thermoanaerobacter* sp. (Toruzyme 3.0 L; after amyloglucosidase treatment)	Buffer	30.0 % (24 h)	40	Mathew and Adlercreutz 2013
β-Arbutin-α-glycoside	Sucrose	β-Arbutin	Amylosucrase from *Deinococcus geothermalis* DSM 11300	Buffer	98 % (−)	35	Seo et al. 2009
Kojic acid glycosides (5-*O*-α-D- and 7-*O*-α-D-glucopyranoside)		Kojic acid	Sucrose phosphorylase from *Leuconostoc mesenteroides*	DMSO:buffer	19.7 % (24 h)	42	Kitao & Serine 1994
Quercetin glycosides (3′-*O*-α-D and 4-*O*-α-D glycopyranoside)		Quercetin	Glucansucrase from *Leuconostoc mesenteroides*	Acetone	23.1 % (5 h)	28	Moon et al. 2007b
Ampelopsin glycosides up to 5 units(4′-*O*-α-D-glycopyranoside as main product)		Ampelopsin	Dextransucrase from *Leuconostoc mesenteroides*	DMSO: buffer	87.3 % (1 h)	28	Woo et al. 2012
Astragalin glycosides (kaempferol-3-*O*-β-D-isomaltoside, 3-*O*-β-D-nigeroside, polymerized 3-*O*-β-D-isomaltooligosaccharides)	Sucrose	Astragalin			24.5 % (5 h)	28	Kim et al. 2012
Ascorbic acid glycosides (2-*O*-α-D-glucopyranosyl L-ascorbic acid as main product)	α-Cyclodextrin	Ascorbic acid	Cyclomaltodextrin glucanotransferase form *Bacillus stearothermophilus*	Buffer	45.6 % (1 h)	60	Aga et al. 1991
Benzoyl glycosides (1-O-benzoyl-α-D-, 2-O-benzoyl-α-D- and 2-O-benzoyl-β-D-glucopyranoside)	Sucrose	Benzoic acid	Sucrose phosphorylase from *Streptococcus mutans*	Buffer	70 % (48 h)	37	Sugimoto et al. 2007

## Glucosidases

Glucosidases, such as α- (EC 3.2.1.20) and β-glucosidases (EC 3.2.1.21), are a group of enzymes that hydrolyze individual glucosyl residues from various glycoconjugates including α- or β-linked polymers of glucose under physiological conditions. However, these enzymes are able to synthesize a broad variety of sugar derivatives under defined conditions in two different manners: transglycosylation or reverse hydrolysis (Park et al. 2005). Active compounds that have been obtained by enzyme-catalyzed glucosidation include vitamin and arbutin derivatives as presented in Table 6. Pharmacological properties of vitamin E can be improved by increasing its water solubility, absorbtivity and stability through glycosylation. A novel water-soluble vitamin E derivative, 2-(α-D-glucopyranosyl)methyl-2,5,7,8-tetramethylchroman-6-ol (TMG) has been synthesized from 2-hydroxymethyl-2,5,7,8-tetramethylchroman-6-ol (TM) and maltose using α-glucosidase from *Saccharomyces* sp. in a transglycosylation reaction (Murase et al. 1998). Anti-oxidant activity of TMG was investigated on peroxidation of phosphatidylcholine-liposomal (PC)-suspension, which is usually adopted as model for cellular biomembranes. TMG showed higher efficacy on lipid peroxidation than ascorbic acid, when peroxidation was provoked by lipid-soluble radical generator such as 2,2′-azobis(2,4-dimethylvaleronitrile (AMVN). Moreover, TMG showed to inhibit cupric ion-induced peroxidation of (PC)-suspension and rat brain homogenate while it delayed the generation of cholesteryl ester hydroperoxides when exposing human plasma to lipid or water-soluble radical generators.

**Table 6:** Glucosidase-catalyzed reactions

Product	Donor	Acceptor	Enzyme	Solvent system	Yield (time)	T (°C)	Reference
4-Hydroxyphenyl-β-isomaltoside	Sucrose	Arbutin	α-Glucosidase from *Saccharomyces cerevisiae*	Buffer	50 % (20 h)	40	Milosavić et al. 2007
Hydroquinone α-D-glucopyranoside	Maltose	Hydroquinone	α-Glucosidase from *Saccharomyces cerevisiae*	Buffer	13 % (20 h)	30	Prodanović et al. 2005
Hydroquinone α-D-isomaltoside					15 % (20 h)		
2-(α-D-Glucopyranosyl)methyl-2,5,7,8-tetramethylchroman-6-ol	Maltose	2-Hydroxymethyl-2,5,7,8-tetramethylchroman-6-ol (vitamin E derivative)	α-Glucosidase from *Saccharomyces* sp.	DMSO	(20 h)	40	Murase et al. 1998
β-D-Glucosyl-(1-6)-arbutin	Cellobiose	Arbutin	β-Glucosidase from *Thermotoga neapolitana*	Buffer	2.8 % (12 h)	80	Jun et al. 2008
β-D-Glucosyl-(1-4)-arbutin							
β-D-Glucosyl-(1-3)-arbutin							

A β-glucosidase from *Thermotoga neapolitana* has synthesized arbutin-β-glycosides to be used as novel skin whitening agents (Jun et al. 2008). β-D-glucosyl-(1–3)-arbutin has been proved to inhibit mushroom tyrosinase and it has been tested on B16F10 murine melanoma cell line showing the strongest inhibitory effect on melanin synthesis in a dose-dependent manner without causing cytotoxicity. β-D-glucosyl-(1–3)-arbutin showed to be a more efficient inhibitor of melanin synthesis compared to arbutin. Similarly, arbutin has been glycosylated by a α-glucosidase from *Saccharomyces cerevisiae* to produce 4-hydroxyphenyl-β-isomaltoside (Milosavić et al. 2007), whose inhibitory capacity on tyrosinase is being investigated. α-Glucosidase from *S. cerevisiae* also catalyzed the synthesis of hydroquinone α-D-glucopyranoside and hydroquinone α-D-isomaltoside (Prodanović et al. 2005). Glycosylation of hydroquinone increased its water solubility and enhanced pharmaceutical properties such as skin whitening and anti-bacterial capacity.

## Laccases

Laccases are dimeric or tetrameric glycosylated proteins, which usually bear four copper atoms per monomer distributed in three redox sites (Gianfreda et al. 1999). These enzymes are able to catalyze the one-electron oxidation of phenols generating phenoxy radicals and simultaneously reducing molecular dioxygen to water (Kudanga et al. 2011). Due to their features, including broad substrate specificity, catalysis in air without using H_2_O_2_, and production of water as only by-product, laccases are considered the ideal green catalysts. Besides catalyzing catabolic and depolymerization processes, based on reaction conditions, these enzymes can also carry out synthetic processes including the oxidization of aromatic compounds followed by heteromolecular coupling with co-substrates or simple oligomerization (Mikolasch and Schauer 2009). The main compounds that have been synthesized by laccase-catalyzed reactions include flavonoids, HCAs, and other phenolics. Conditions of their production are described in the following sections and summarized in Table 7.

**Table 7:** Laccase-catalyzed reactions

Product	Donor	Acceptor	Enzyme	Solvent system	Yield (time)	T (°C)	Reference
Caffeic acid-chitosan	Caffeic acid	Chitosan	Laccase from *Trametes versicolor*	Phosphate buffer	– (24 h)	30	Božič et al. 2012a
Gallic acid-chitosan	Gallic acid				– (24 h)		
Quercetin-chitosan	Quercetin	Chitosan			– (24 h)		Božič et al. 2012b
Gallic acid-chitosan	Tannic acid						
Starch–sodium lignosulfonate graft copolymers	Sodium lignosulfonate	Starch		Sodium acetate	– (4 h)	30	Shogren and Biswas 2013
3,3,5,5-Tetramethoxy biphenyl-4,4-diol	2,6-Dimethoxyphenol	2,6-Dimethoxyphenol	Laccase from *Trametes pubescens*	Ethyl acetate	– (24 h)	28	Adelakun et al. 2012a
				Acetone	– (24 h)		
Ferulic acid dimers (5-β, β-β)	Ferulic acid	Ferulic acid		Ethyl acetate or dioxane or ethanol	– (24 h)		Adelakun et al. 2012b
Poly 8-hydroxyquinoline	8-Hydroxyquinoline	8-Hydroxyquinoline		Acetone	76 % (24 h)	30	Ncanana and Burton 2007
Resveratrol trans-dehydrodimer	Resveratrol	Resveratrol		Ethyl acetate	18 % (4 days)	45	Nicotra et al. 2004
			Laccase from *Myceliophtora thermophyla* (supported on glass beads)	n-Butanol	31 % (4 days)		
Ethyl-ferulate-chitosan	Ethyl ferulate	Chitosan	Laccase from *Myceliophtora thermophyla*	Phosphate buffer	(4 h)	30	Aljawish et al. 2012
Ferulic acid-chitosan	Ferulic acid				(4 h)		
Poly-catechin	(+) - Catechin	(+) - Catechin		Acetone	95 % (24 h)	RT	Kurisawa et al. 2003b
Poly-rutin	Rutin	Rutin		Methanol	79 % (24 h)	RT	Kurisawa et al. 2003a
Oligorutin	Rutin	Rutin	Laccase from *Pycnoporus coccineus*	Glycerol/ethanol/buffer	67 % (24 h)	RT	Uzan et al. 2011
			Laccase from *Pycnoporus sanguineus*				

The anti-oxidant activity of flavonoids derives from the B-ring, which is important for the H-transfer, and 2–3 double bond ensuring electron delocalization. Moreover, in vitro studies have demonstrated the importance of the 3-OH group for the anti-oxidant capacity. Rutin has been oxidized by a laccase from *Myceliophtora thermophyla* to produce flavonoid polymers (Kurisawa et al. 2003a). The same result was achieved by using *Pycnoporus coccineus* and *Pycnoporus sanguineus* laccases as biocatalysts. Oxidized poly-rutin showed enhanced anti-oxidant, anti-inflammatory, and anti-aging capacities compared to the rutin monomer (Uzan et al. 2011). Enzymatic oxidation of catechin was also performed by a laccase from *M. thermophyla* producing poly-catechin with greater superoxide scavenging and inhibitory capacity of xanthine oxidase (Kurisawa et al. 2003b). Laccase-catalyzed oxidation has been applied in order to enhance the anti-oxidant property of FA. Two dimeric products, β-5 and β-β, were obtained by oxidation of FA in organic media using a laccase from *Trametes pubescens* (Adelakun et al. 2012b). Reaction was performed in a biphasic system, as the concentration of ethyl acetate increased, and in monophasic system using dioxane or ethanol as co-solvents. The β-5 dimer showed higher anti-oxidant capacity than FA evaluated by DPPH and Trolox equivalent antioxidant capacity (TEAC) assays. Different oxidized products of HCAs were used to improve anti-oxidant and anti-microbial activities of polymers, such as chitosan. A laccase from *M. thermophyla* was used to functionalize chitosan with oxidated FA and ethyl-ferulate (Aljawish et al. 2012). Both derivatives showed higher anti-oxidant activity than the substrates, especially the FA chitosan. The same strategy was applied to functionalize chitosan with CA using a laccase from *Trametes versicolor*, obtaining a functionalized polymer with higher anti-oxidant and anti-microbial activity than the substrates (Božič et al. 2012b). These results indicated that the addition of an H-atom donating group, produced by laccase-mediated oxidization, could generate a good chain breaking anti-oxidant. Laccase-mediated oxidation is proved to be a good strategy to develop functionalized polymers with enhanced anti-oxidant and anti-microbial activities.

Oxidation of tannic acid by a laccase from *T. versicolor* resulted in a variety of products including gallic acid, gallic acid dimers, partially gallic acid-esterified glucose, and glucose, while oxidation of quercetin offered an oligomeric derivative (Božič et al. 2012a). Both oxidative products of gallic acid and quercetin showed higher anti-oxidant activity than the origin compounds. Furthermore, tannic acid or quercetin was used to functionalize chitosans by laccase without organic or acidic solvents. Both chitosan derivatives exhibited amplified radical scavenging ability and anti-microbial activity compared to the untreated chitosans. The laccase grafting method was also applicable to other phenolic compounds, as in the case of graft copolymers of starch with lignosulfonates (Shogren and Biswas 2013). Enzymatic polymerization of 8-hydroxyquinoline was achieved by using a laccase from *T. pubescens* (Ncanana and Burton 2007). Oxidization of 8-hydroxyquinoline was established to generate aromatic radicals which combined to form a polymeric product with a powerful anti-oxidant capacity and anti-radical efficiency. Laccase-mediated oxidization was also performed in organic solvents, due to their advantages as medium in biocatalysis. Oxidation of 2,6-dimethoxyphenol by *T. versicolor* laccase was investigated in biphasic or monophasic systems, leading the formation of a dimeric product with anti-oxidant capacity twofold higher than the substrate. The dimer production was increased in the monophasic solvent using acetone as co-solvent, while its production increased as the concentration of ethyl acetate was increased to 90 % in the biphasic system. Organic solvents were also applied in the synthesis of resveratrol dimers catalyzed by laccases from *M. thermophyla* and *T. pubescens* (Nicotra et al. 2004). *M. thermophyla* laccase-catalyzed dimers were obtained in butanol saturated with buffer; and resveratrol dimers catalyzed by *T. pubescens* laccase were synthesized using a biphasic system of ethyl acetate and buffer. The products may serve as lead for the development of new drugs and as nutraceuticals, showing anti-oxidant activity comparable to resveratrol and its analogs.

## Conclusions

A large variety of compounds with potential cosmeceutical application can be obtained through biotechnological processes. The reported examples of enzymatic synthesis or modification of natural compounds so far exploited highlight the possibility of developing biologically active ingredients with anti-oxidant, anti-aging, anti-microbial, anti-wrinkling, photoprotective, or skin-whitening properties. The use of esterases (such as lipases, feruloyl esterases, tannases), transferases, glucosidases, proteases, and laccases allows the modification of target compounds under mild conditions, maintaining their biological activity and avoiding the formation of by-products. These advantages fit the increasing demand for natural cosmetics, boosting eco-friendly design and production of active compounds in order to replace chemical processes currently used.
